# Is type of depressive symptoms associated with patient-perceived need for professional psychological care in depressed individuals with diabetes?

**DOI:** 10.1371/journal.pone.0212304

**Published:** 2019-02-14

**Authors:** L. J. van der Donk, J. Fleer, R. Sanderman, P. M. G. Emmelkamp, T. P. Links, K. A. Tovote, M. J. Schroevers

**Affiliations:** 1 Department of Health Psychology, University Medical Center Groningen, University of Groningen, Groningen, the Netherlands; 2 Department of Psychology, Health and Technology, University of Twente, Enschede, the Netherlands; 3 Department of Clinical Psychology, University of Amsterdam, Amsterdam, the Netherlands; 4 Department of Endocrinology, University Medical Center Groningen, University of Groningen, Groningen, the Netherlands; Arizona State University, UNITED STATES

## Abstract

**Aims:**

The objective of this study is to investigate whether type of depressive symptoms (i.e. cognitive-affective or somatic) is related to a patient-perceived need for professional psychological care in individuals with diabetes.

**Methods:**

In total 2266 participants were recruited as part of the screening procedure for a multi-center randomized controlled trial on the treatment of depressive symptoms among individuals with diabetes. Individuals were invited to complete Beck Depression Inventory-II (BDI-II). Patients with elevated depressive symptoms (BDI-II ≥14) were interviewed about their psychological care need. Based on their care needs patients were categorized into: unmet need, no need, met need and unclear need. These groups were compared on type of depressive symptoms, as categorized into cognitive-affective symptoms and somatic symptoms.

**Results:**

568 eligible individuals had elevated depressive symptoms, of whom 519 were reached. Among these depressed individuals, 19.7% (102 of 519) had an unmet need for psychological care. Participants with an unmet need were younger (*p*<0.001) and had higher total depression scores compared to the group with no need (*p*<0.001). They also scored higher on cognitive-affective symptoms (*p*<0.001), whereas somatic symptoms did not significantly differ (*p* = 0.232). Logistic regression revealed that cognitive-affective symptoms predicted an unmet need (*p* = 0.001). However, overall predictive capacity of type of depressive symptoms on care needs was weak.

**Conclusions:**

Cognitive-affective symptoms of depression—but not somatic symptoms—were associated with an unmet need for psychological care among depressed individuals with diabetes. Future research is needed to reveal better predictors explaining the discrepancy between distress and low care needs in order to optimize screening procedures.

## Introduction

Depressive symptoms are a common comorbidity in diabetes mellitus type 1 and 2, with a prevalence as high as 31% [1] which is two to three times higher compared to individuals without diabetes [2,3]. This comorbidity is associated with poorer health outcomes in terms of medication and diet regimen nonadherence, poorer glycemic control, increased morbidity, increased mortality rates and increased healthcare costs [4–6]. Psychological interventions have been found to be effective in the treatment of depressive symptoms in persons with diabetes [7], with respect to both psychological outcomes (i.e. reduced levels of depressive symptoms and anxiety) and diabetes-related outcomes (i.e. Hba1c). Improvement of psychological outcomes and recovery is associated with significant reductions in work disability and healthcare costs [8] and has been found to be cost-effective [9]. These findings stress the importance of early detection and treatment of depressive symptoms among individuals with diabetes.

International guidelines advocate routine screening in order to identify people in need of psychological help [10,11] and refer them for effective treatment [5]. Frequently used screening instruments for detecting distress or elevated depressive symptoms among chronically ill patients include the Distress Thermometer (DT), Beck Depression Inventory-II (BDI-II) and Centre for Epidemiologic Studies Depression Scale (CES-D) [12,13]. Screening is however only effective when followed by uptake of treatment [5]. Currently, there are three usual ways regarding depression management for patients with diabetes [14]. In primary care settings, the majority of patients receive antidepressant medication, which have been found to be effective in reducing depressive symptoms [14]. Another effective treatment option is psychotherapy, which includes a variety of treatments such as cognitive behavioral therapy and problem-solving therapy. A final depression treatment option involves collaborative care, which combines antidepressant medication and psychotherapy.

Screening may be a useful tool for identifying depressed persons with diabetes, because previous research has shown that the uptake of depression care is low, due to poor depression recognition by healthcare professionals in standard care [15–17]. Another possibility for the low uptake of depression care is that many depressed persons with diabetes do not have a need for psychological care. One study that investigated care needs in individuals with diabetes [18] showed that only a minority (34.6% of 104 participants) of depressed or distressed persons desired referral for psychosocial care. The main self-reported reasons for these participants to refuse referral were lack of interest and no time. Another recent study among persons with diabetes screened for participation in a trial and found that only 21% of the 130 individuals with elevated scores was willing to participate [19]. Main reported reasons for non-participation included not being interested in receiving help and finding it too strenuous. These findings resemble results from several studies in individuals with cancer, in which an increasing number of researchers have been examining perceived need for psychosocial services. Findings showed that despite elevated levels of distress, only a minority (ranging from 14% to 35%) perceived an unmet need for professional psychological care [12,20–24]. The most commonly reported reasons for not perceiving a need for care involve the preference for doing it themselves [20,23–26] and the reliance on (informal) social support [20,22,23]. It should be noted in all the above-mentioned studies that the reasons for declining help were self-reported by patients during the intake. It is therefore possible that there were underlying reasons for reporting ‘lack of interest’ that were not further discussed by the interviewer. Possible underlying reasons, of which patients may be aware or not, may include a lack of perceived benefit of depression care, stigmatization for receiving psychological care, a low burden of symptoms, or perceiving sufficient coping and social resources to manage the symptoms on their own [20,23–26]. These results suggest that despite elevated levels of distress or depressive symptoms, only a minority of individuals with chronic somatic diseases (including diabetes) perceive a need for professional psychological care.

One of the reasons for a low need for care might be that individuals with diabetes do not recognize that (some of) their symptoms are attributable to depression. That is, the construct of depression is very heterogeneous [27,28] consisting of cognitive, affective and somatic symptoms [29–31]. The somatic symptoms of depression may overlap with the somatic symptoms of diabetes (e.g. fatigue, weight loss), making it difficult for individuals with diabetes to differentiate whether their complaints originate from the disease or from depression. Indeed, previous research has shown that the presence of somatic symptoms is poorly recognized as being part of depression by the physician but also by patients themselves due to the tendency to attribute physical complaints more to a physical illness than to a psychological cause [32]. Moreover, a study in primary care found that the presence of somatic symptoms resulted in a decreased likelihood of initiating depression treatment [33]. Possibly, persons with somatic symptoms of depression believe that receiving psychological care may not be helpful for alleviating somatic symptoms and therefore decline psychological help. Therefore, it could be hypothesized that persons with high cognitive-affective symptoms, with or without additional somatic symptoms, are more likely to recognize their depressive symptoms and perceive a need for psychological care than those with lower levels of cognitive-affective symptoms.

To our knowledge, this study is the first to explore whether type of depressive symptoms is related to a need for psychological care among individuals with diabetes. We hypothesized that individuals with high levels on cognitive-affective symptoms are more likely to perceive an unmet need for psychological care, compared to those with low levels of these symptoms. No such association is expected for individuals with high levels of somatic symptoms.

## Material and methods

### Sample and procedure

Participants in this study were recruited as part of the screening procedure for a multicenter RCT on the effectiveness of treating depressive symptoms in individuals with diabetes [34]. The study was approved by the Medical Ethical Committee of the University Medical Center Groningen. Persons in treatment for diabetes (both type 1 and type 2) at one of the participating outpatient clinics were approached from June 2011 to February 2013 and invited to fill in a self-report screening questionnaire including age, gender and BDI-II. Inclusion criteria for the current study were: aged between 18 and 70 years, being able to read/write Dutch and having elevated depressive symptoms (as indicated by BDI-II score of ≥14). Exclusion criteria were: pregnancy, severe psychiatric comorbidity, acute suicidal ideation and unstable treatment with an antidepressant in the last 2 months prior to inclusion in the study.

Persons with elevated depressive symptoms were contacted by phone and consequently invited for a face-to-face intake. They were first informed about their elevated depressive symptoms on the questionnaire and then asked whether this reflected their actual mood. Consequently, we asked whether they were currently receiving psychological help and if not, they were asked: *“Are you interested in receiving psychological help for your depressive complaints*?*“*. Answers to this question were summarized and written down. If participants declined psychological help, they were asked for the reason for this (by using an open question). Those who accepted psychological help were given more information about the participation in the RCT or were referred for further treatment. More detailed information about the RCT procedure is described elsewhere [34].

### Measures

**Fig 1:**
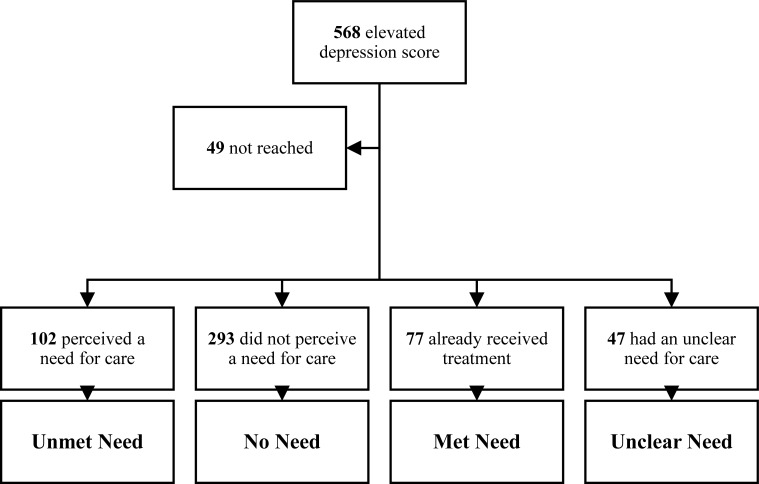
Flowchart of 519 depressed persons with diabetes categorized into four perceived care need groups.

The main outcome is depressive symptoms, measured by BDI-II. This is a self-report questionnaire with 21 items. Each item contains 4 answer categories scaled from 0 to 3, resulting in a total score between 0 and 63, with higher scores indicating higher levels of depressive symptoms. Cronbach’s alpha in our sample was 0.83. For the purpose of our study, we divided 21 items into cognitive-affective domain comprising of item 1–14 (e.g. worthlessness or loss of pleasure) and somatic domain with items 15–21 (e.g. fatigue or concentration difficulty), based on previous models [29,35]. To verify these domains in our diabetes sample, principal component analysis was executed and suggested two components. With the exception of the item “irritability” (scoring on both domains), the principal component analysis displayed a close-to-perfect fit regarding the above-described two domains. For perceived need for psychological care, data were retrieved from the written summary regarding the need question during the interview. Answers of those who declined psychological help were later divided into three different categories by two of the authors (i.e. LJD and KAT). In total, four groups were created based on participants’ need for psychological care as displayed in Fig 1: (1) unmet need for psychological care, (2) no need, (3) met need, (4) unclear need. The ‘unmet need’ group consists of individuals who desired psychological help for their complaints. The ‘no need’ group involved those not perceiving a need. The ‘met need’ group comprised persons that already received psychological help. The ‘unclear need’ group were individuals with inconsistent or lacking information regarding their need (e.g. those who reported that they maybe have a need but not at the moment or those reporting to have no time for help).

### Statistical analyses

For statistical analyses, SPSS 23.0 was used. Descriptive statistics for demographic variables were calculated (i.e. means and standard deviation) by means of ANOVAs and chi-square tests. Post-hoc tests were used to reveal possible differences among the groups. In addition, the four care need groups were compared on total BDI-II scores and subscales (i.e. cognitive-affective and somatic) using ANOVAs. Because we were interested in the difference between those with an unmet need versus those with no need, a logistic regression was executed to examine both domains as predictors for unmet need for care (versus no need) with adjustment for covariates. Interaction terms between the two domains were also added. All predictors were standardized prior to analysis by substraction of the mean and dividing this by the standard deviation.

## Results

In total 2266 individuals with diabetes were consecutively screened, with 562 eligible persons having elevated depressive symptoms (24.8%). Additionally, 6 individuals were referred by their physician resulting in 568 depressed patients with diabetes. Of those 568, 49 individuals could not be reached for the telephonic interview after several attempts. Therefore 519 participants remained (all Caucasian), which were divided in four groups based on their need for care (see Fig 1). Mean age was 55.2 years with 50.4% being male.

### Sample characteristics

Of the 519 participants, 102 had an ‘unmet need’ (19.7%), 293 had ‘no need’ (56.5%), 77 had a ‘met need’ (14.8%) and 47 (9.1%) had an ‘unclear need’. In general, the ‘no need’ group was significantly older compared to the ‘unmet need’ group and the ‘met need’ group. No differences in gender were found between the four groups (Table 1).

**Table 1:** Sample characteristics of 519 depressed persons with diabetes categorized into four groups based on their perceived need for psychological care.

	Unmet need	No need	Met need	Unclear need	*P*-value
N	102	293	77	47	
Age (years)	53 ±11.9	57 ±11.0	51 ±12.9	54 ±11.8	**<0.001**
Gender (% male)	51.0	51.5	42.9	55.3	0.499

For age, P-value is derived from ANOVA; for gender, P-value is from χ^2^-test. Data are means and ± standard deviation for age and percentages of individuals for gender.

### Comparison between four care need groups

The ‘unmet need’ group had higher total depression scores compared to the ‘no need’ group (*p*<0.001) and scored also higher on the cognitive-affective domain (*p*<0.001). No significant differences were found on the somatic domain between the four groups (*p* = 0.232) (Table 2).

**Table 2:** Comparison of 519 depressed persons with diabetes on total BDI-II scores and subscales divided in four groups based on perceived need.

		Unmet need	No need	Met need	Unclear need	*P*-value
BDI score-II		23.8 ±8.5	20.3 ±6.6	23.9 ±9.7	24.4 ±10.3	**<0.001**
	Cognitive-affective subscale	14.5 ±6.4	11.5 ±5.5	15.0 ±7.3	14.7 ±8.6	**<0.001**
	Somatic subscale	9.3 ±3.4	8.8 ±2.8	9.0 ±3.4	9.7 ±3.2	0.232

BDI-II = Beck Depression Inventory-II

### Comparison between unmet need and no need for care

Logistic regression was conducted with demographic variables (i.e. age and gender) as covariates. Table 3 shows regression coefficients, Wald Statistics, odds ratios, and 95% confidence intervals for odds ratios for each predictor. A test of the full model against a constant only model was statistically significant, indicating that predictors reliably distinguished between having an unmet need versus having no need (χ^2^ = 22.98, *p*<0.001 with df = 4). Wald criterion indicated that only the cognitive-affective subscale significantly contributed to the prediction (*p* = 0.001), after adjustment for age and gender. The odds ratio of 1.579 indicates that for one standard deviation increase in cognitive-affective score, a 58% increase in odds of having an unmet care need is expected. The somatic subscale was not significant in predicting unmet need (*p* = 0.400) and neither was the interaction (*p* = 0.582).

**Table 3:** Logistic regression predicting (no) need for care in the subgroup of 395 depressed persons with diabetes who endorsed ‘unmet need’ or ‘no need’.

Variables	B	Wald Chi-Square	Odds ratio	95% Confidence Interval for Odds Ratio		*P*-value
				*Lower*	*Upper*	
*Step 1*						
Age	-0.322	6.214	0.725	0.563	0.934	**0.013**
Gender	0.074	0.359	1.077	0.845	1.372	0.549
Cognitive-affective subscale	0.457	10.938	1.579	1.204	2.070	**0.001**
Somatic subscale	0.116	0.709	1.123	0.857	1.473	0.400
*Step 2*						
Cognitive-affective * somatic	0.071	0.303	1.074	0.833	1.384	0.582

Nagelkerke’s R^2^ of 0.086 indicated that little variance is explained in the model. Prediction success overall of the model was 74.1% (96.5% for no need and 7.3% for unmet need), suggesting that the model with predictors has good specificity properties (i.e. detecting no need cases), but very low sensitivity in terms of correctly predicting an unmet need case. Interestingly, the overall prediction success of the null model (without predictors, in which all cases are assigned to the largest group) was 74.9% which is almost similar to the prediction model. In other words, adding these predictors to the model did not result in an overall better predictive capacity.

## Discussion

The current study examined whether need for care in depressed individuals with diabetes is related to the type of depressive symptoms (i.e. cognitive-affective and somatic) they are experiencing. As hypothesized, individuals with diabetes reporting higher levels of cognitive-affective symptoms were significantly more likely to report an unmet need for psychological care, compared to those reporting lower levels of these symptoms. Somatic symptoms were not significantly associated with perceiving an unmet need for psychological care. Possibly, individuals with high levels of somatic symptoms are less likely to recognize and link these somatic symptoms to being depressed and the need for psychological care. It could also be the case that they think the offered psychological treatment would not meet their somatic complaints and/or is not effective for treating somatic symptoms. Alternatively, it can be reasoned that those individuals who scored high on somatic items were not depressed and therefore did not report a greater need for psychological care. Rather they were experiencing somatic complaints related to their physical health condition [13,27]. It has been debated whether somatic symptoms should be included in the measurement of depressive symptoms in those with a somatic condition. A study on depression screening in diabetes patients using the BDI concluded that “somatic items did not interfere with the screening utility of the total BDI” [36]. In fact, the ROC curve based on the total BDI was slightly better than that based on the cognitive-affective symptoms alone. In addition, it may be useful to also take into account the specific nature and type of depressive symptoms when relating to diabetes outcomes, as research has shown that different subtypes of depression differentially affect diabetes outcomes [37]. With respect to need for care, our results suggest that such a need is mainly related to the presence or absence of cognitive and affective symptoms.

Even though our hypotheses are supported, it should be noted that the predictive capacity of the model indicated that overall, type of depressive symptoms is not a strong predictor for need for psychological care. Two other studies have drawn similar conclusions. A recent study among cancer patients found that distress, anxiety and depression predicted only little variance of the need for care, suggesting that having an unmet need might be explained by other variables [23]. For instance, stigmatization for receiving professional psychological care may form a barrier for accepting help. Another study in the general population did not find evidence that type of depressive symptoms was a good predictor for treatment seeking either [38]. In our study only one in five of the depressed individuals with diabetes had an unmet need for psychological care. This is an important finding, as it gives information about the efficiency of depression screening to identify patients with a need for care. Interestingly, similar percentages have been found in other patients groups such as cancer patients [12,20–24]. These low uptake rates question the utility of routine screening on depressive symptoms among individuals with diabetes. Considering the importance of identifying individuals with psychological symptoms who are in need of psychological care and may benefit from such care, alternative ways for efficiently identifying those with an unmet need for care should be considered.

One possible way may be complementing a distress or symptom questionnaire with a list of problems such as the Problem Areas in Diabetes (PAID) [39]. The PAID is a diabetes specific questionnaire and may give additional insights into determinants of need for care, because research in oncology has shown that (in particular practical and emotional) problems are a strong predictor for referral wish [22]. Another alternative, already introduced in the oncology setting, involves a simple help question in order to identify care needs among distressed individuals [23]. Both the PAID and the help question might be beneficial in terms of better revealing care needs in individuals with diabetes.

Nevertheless, we would like to point out that merely focusing on those with an unmet need for care is insufficient, since depression is a severe condition with patients experiencing high levels of distress and comorbid negative health outcomes including poor glycemic control [5]. It is essential that individuals not expressing a need for psychological care are also taken into account. For instance, it might be beneficial to create more awareness (e.g. psychoeducation) about the symptoms these individuals are experiencing and how to interpret these symptoms (e.g. providing insight that somatic complaints may also arise from a depressed mood). Otherwise, strategies may be implemented aimed at enhancing motivation in individuals with no need for psychological care such as Motivational Interviewing (MI). MI has already been found to increase health screening uptake [40] and may therefore also have the potential to increase motivation among those who were earlier unwilling to accept psychological help.

Despite our finding of little predictive value for somatic symptoms regarding need for care, somatic symptoms should not be overlooked. Instead, it should be considered that somatic symptoms can originate from diabetes-related complaints as well as depression. In general, our results stress the importance of a multidisciplinary approach, given the fact that it is not always possible to distinguish for patient or doctor whether complaints originate from diabetes or depression.

Our study contains several limitations. First, it should be taken into account that some individuals with elevated depressive symptoms included in this study are false positives, meaning they were not depressed and therefore did not report a need for treatment. This was also concluded by a systematic review [13], but this problem cannot be addressed by the current design of our study. However, a study in people with diabetes found that the positive predictive value of the BDI-II was fairly good (0.80 with prevalence of 37%) [36]. As a total BDI-II score is used in screening practice to further discuss the problems and possibly refer someone for additional help, we were interested in examining whether type of depressive symptoms is related to a need for care in all patients with elevated BDI-II scores. Secondly, the measure of need for psychological care was dichotomized into “unmet need” versus “no need” whereas it is more likely to be a continuous construct. Besides, when an individual indicated to perceive no need for psychological treatment, the underlying reason for this was not specified. In addition, other psychological measures such as anxiety, quality of life or distress were not taken into account. All of these variables might determine whether an individual perceives a need for care. Regarding the sample description, information is missing about marital status (or social support), educational level and type of diabetes. These could not be obtained since the data were collected as part of a screening procedure.

More research is needed to better understand why people with depressive symptoms generally have a low need for care. It seems that the quantity of symptoms or the type of (depressive) symptom is not conclusive to determine whether one perceives an unmet need. Alternatively, possibly the way individuals perceive the meaning and impact of the symptoms on their life and the way they are able to cope adaptively with these symptoms or not, has a greater impact on their need for care. More insight into determinants for care needs may be gained by conducting qualitative research on this topic. This future research should reach beyond the reasons for declining help as identified by previous (cancer) research, including a wish to cope with symptoms on their own [20,23–25]. Instead, it should rather focus on the appraisal and coping of the depressive symptoms. Such knowledge can be used to optimize screening in individuals with diabetes and the uptake of psychological care.

Our results suggest that a greater experience of cognitive-affective symptoms was associated with perceiving an unmet need. Yet overall, the level and type of depressive symptoms were poor predictors of persons’ need for psychological care. Other yet to be discovered factors might influence whether or not individuals perceive a need for additional psychological care. Ultimately, more research is needed in order to determine the underlying reasons and mechanisms for participants’ unwillingness to accept psychological help.

## Supporting information

S1 Dataset SPSS data file. (SAV)
